# Factors Associated with Anemia among Pregnant Women of Underprivileged Ethnic Groups Attending Antenatal Care at Provincial Level Hospital of Province 2, Nepal

**DOI:** 10.1155/2021/8847472

**Published:** 2021-02-12

**Authors:** Umesh Kumar Yadav, Prabesh Ghimire, Archana Amatya, Ashish Lamichhane

**Affiliations:** ^1^Department of Community Medicine and Public Health, Maharajgunj Medical Campus, Institute of Medicine, Tribhuwan University, Kathmandu, Nepal; ^2^Feed the Future Innovation Lab for Nutrition, Kathmandu, Nepal

## Abstract

**Background:**

This study aims at determining the factors associated with anemia among pregnant women of underprivileged ethnic groups attending antenatal care at the provincial level hospital of Province 2.

**Methods:**

A hospital-based cross-sectional study was carried out in Janakpur Provincial Hospital of Province 2, Southern Nepal. 287 pregnant women from underprivileged ethnic groups attending antenatal care were selected and interviewed. Face-to-face interviews using a structured questionnaire were undertaken. Anemia status was assessed based on hemoglobin levels determined at the hospital's laboratory. Bivariate and multiple logistic regression analyses were used to identify the factors associated with anemia. Analyses were performed using IBM SPSS version 23 software.

**Results:**

The overall anemia prevalence in the study population was 66.9% (95% CI, 61.1–72.3). The women from most underprivileged ethnic groups (Terai Dalit, Terai Janajati, and Muslims) were twice more likely to be anemic than Madhesi women. Similarly, women having education lower than secondary level were about 3 times more likely to be anemic compared to those with secondary level or higher education. Women who had not completed four antenatal visits were twice more likely to be anemic than those completing all four visits. The odds of anemia were three times higher among pregnant women who had not taken deworming medication compared to their counterparts. Furthermore, women with inadequate dietary diversity were four times more likely to be anemic compared to women having adequate dietary diversity.

**Conclusions:**

The prevalence of anemia is a severe public health problem among pregnant women of underprivileged ethnic groups in Province 2. Being Dalit, Janajati, and Muslim, having lower education, less frequent antenatal visits, not receiving deworming medication, and having inadequate dietary diversity are found to be the significant factors. The present study highlights the need of improving the frequency of antenatal visits and coverage of deworming program in ethnic populations. Furthermore, promoting a dietary diversity at the household level would help lower the prevalence of anemia. The study findings also imply that the nutrition interventions to control anemia must target and reach pregnant women from the most-marginalized ethnic groups and those with lower education.

## 1. Background

Anemia, a condition with low blood hemoglobin concentration and/or red blood cells (RBCs), is a global public health problem that mostly affects low- and middle-income countries (LMICs) [1]. Anemia in pregnancy poses a greater risk for low birth weight, preterm birth, and perinatal and neonatal mortality [2]. Besides, the severity of anemia is associated with higher rates of maternal mortality [3]. Anemia affects over half a billion women of reproductive age worldwide. It is estimated to affect 38% (32.4 million) of pregnant women globally with highest prevalence in the World Health Organization (WHO) regions of South-East Asia (48.7%) and Africa (46.3%) [4]. Evidence from various low- and middle-income countries suggests that anemia is disproportionately concentrated in the low socioeconomic group [5] with poorest, ethnically disadvantaged, and least educated at the greatest risk.

In Nepal, 41 percent of reproductive age women are anemic with highest prevalence in Province 2 (58%) [6]. The anemia prevalence in women is disproportionately higher among Terai Dalit (so-called untouchable Terai inhabitants), Terai Janajati (indigenous Terai people), Terai/Madhesi Brahmin/Chhetri (upper caste Terai inhabitants), and Muslims [7]. These ethnic groups constitute over three-fourths of the population in Province 2 [8]. Yet, they are historically marginalized and have performed poorly as measured by indicators of poverty, health, nutrition, education, and women's empowerment. The utilization of health services by Terai Dalit, Terai Janajati, Muslim, and Madhesi is consistently poor for the past many years [7]. Furthermore, studies have also shown that Terai women are more likely to be anemic than women in other regions [9–11].

Reducing anemia prevalence among women and children has been a longstanding priority for Nepal. The Government of Nepal is determined to meet various global and national targets for anemia reduction based on its commitments towards National Strategy for Control of Anemia among Women and Children in Nepal 2002, Multi-Sectoral Nutrition Plan II (2018–2022), Sustainable Development Goals (SDG) 2030, and World Health Assembly Global Nutrition Targets of achieving 50% reduction in anemia among women of reproductive age by 2025. In this context, the Ministry of Health and Population has made many remarkable efforts to reduce anemia in women through activities such as universal, daily iron-folic acid (IFA) supplementation to pregnant and lactating women, deworming program to reduce the burden of parasitic infections, and mandatory fortification of wheat flour with iron, folic acid, and vitamin A [12, 13]. Yet, anemia is still a public health problem in Nepal. This is more challenging as women from the poorest and marginalized groups are the most affected [7].

The 2030 agenda for sustainable development has urged countries to place special emphasis on those left furthest behind and the most excluded with a strong focus on leaving no one behind. This agenda also requires the country to reach its goals and targets for all people and all segments of the society [14]. The national strategy for reaching the unreached (2016–2030) has also clearly highlighted that the reduction of health and nutrition inequalities and achievement of a universal health coverage in the country can only be realized if unreached populations are systematically targeted [15]. In this context, nutrition programs, actions, and strategies are necessary that target the most vulnerable communities bearing a disproportionate burden of anemia [11]. This warrants an appropriate investigation among marginalized ethnic groups and those furthest behind. Although a wide range of research studies in Nepal have attempted to examine the factors associated with anemia, majority of these analyses are specifically limited to the general population such as women, children, and adolescents. Disaggregated analysis involving key subpopulations particularly the ethnic groups which are underprivileged and have greater vulnerability to anemia has not yet been available for Nepal. It is against this background that this study was conducted. The study aimed at assessing the factors associated with anemia among pregnant women of underprivileged ethnic groups who attended antenatal care at the provincial level hospital of Province 2. A better understanding of the local determinants of anemia is considered crucial to identify and implement evidence-based and contextually appropriate strategies [5].

## 2. Methods

### 2.1. Study Design and Settings

This was a hospital-based cross-sectional study conducted at Janakpur Provincial Hospital, located at the Janakpurdham, the capital of Province 2, Nepal. Province 2, one of the seven provinces, consists of eight districts that extend in the southeastern flat plains (Terai region) of Nepal. Despite its ecological richness, Province 2 fares poorly in various socioeconomic and health indicators including but not limited to literacy, teenage pregnancy, nutrition, contraceptive use, immunization coverage, and exposure to domestic violence. As per the Nepal Demographic Health Survey, the prevalence of anemia among women of reproductive age was reported the highest in Province 2 [6]. Janakpur Provincial Hospital is the largest referral level public hospital in Province 2 offering a wide range of healthcare services including antenatal, maternal, and newborn care. This hospital receives patients and clients largely from Dhanusha and surrounding four districts (Siraha, Mahaottari, Sarlahi, and some parts of Sindhuli).

### 2.2. Study Population

This study was carried out among pregnant women who attended antenatal care (ANC) in the Janakpur Provincial Hospital. Women in the second and third trimester of pregnancy and belonging to the underprivileged ethnic groups were included in the study. However, women with severe obstetric complications and those with the history of intake of steroidal drugs were excluded from the study. The underprivileged groups in this study constituted of Terai Dalit, Terai Janajati, Muslim, and Madhesi. These groups have historically suffered oppression, discrimination, and social-segregation and are politically, economically, and socially backward [16, 17]. They are often unable to enjoy social services and facilities and face significant inequalities in the utilization of health care [7, 18]. Terai Dalits are ascribed the lowest position in the caste-ethnicity hierarchical structure and represent the most depressed category among all ethnic groups. They have suffered from acute landlessness and caste-based discrimination, including untouchability [16, 19]. Madhesi caste group grips a relatively better advantage as compared to the other three groups [20].

### 2.3. Study Design and Sampling Procedure

The sample size was calculated using Epi Info StatCalc software assuming 95% level of confidence, 0.06 margin of error, and 57.8% anemia prevalence among reproductive age women of Province 2 [6]. A minimum sample of 261 was estimated and it was increased to 287 considering the nonresponse rate of 10%. The study participants attending antenatal care between 10 am and 4 pm were consecutively enrolled until the planned sample size was achieved.

### 2.4. Data Collection

A structured questionnaire was developed based on the study objectives. The standard food and dietary recall questionnaire developed by Food and Nutrition Technical Assistance (FANTA) Project was used to assess the dietary diversity status [21]. The questionnaire was divided into four broad sections: sociodemographic information, preventive health practices, dietary practices, and hemoglobin level. Data collection was carried out between November and December 2017 using a face-to-face interview with the pregnant women at the antenatal care (ANC). Interviews were conducted in a separate room after the participants received their antenatal services. The interview was administered by the first author who could speak both Nepali and Maithili (local) languages. In order to determine the status of anemia, blood was drawn from each participant with the help of a certified lab technician. The blood samples were collected and tested in the laboratory of Janakpur Provincial Hospital. The collected blood samples were checked for hemoglobin level using the cyanmethemoglobin method.

## 3. Measurement of Variables

### 3.1. Anemia

The pregnant women were considered anemic if they had a hemoglobin concentration less than 11.0 g/dl [22]. Anemia was further categorized as mild (hemoglobin = 10.0–10.9 g/dl), moderate (hemoglobin = 7.0–9.9 g/dl), and severe (hemoglobin <7.0 g/dl) [22].

### 3.2. Dietary Diversity Status

This was a dichotomous indicator of whether or not women have consumed at least five out of ten defined food groups within 24 hours. In order to determine this, we used a 24-hour dietary recall questionnaire gathering information on all foods and beverages consumed by the participants in the previous day and night. The foods consumed were aggregated into 10 recommended food groups: starchy staples, pulses (beans, peas, and lentils), and nuts and seeds; dairy; meat, poultry, and fish; eggs and dark green leafy vegetables; vitamin A-rich fruits and vegetables; other vegetables; and other fruits. For each food group the pregnant women consumed from, a score of 1 was provided and 0 otherwise. The scores from all ten food groups were added to obtain the total dietary diversity score ranging from 0 to 10. The dietary diversity score thus obtained was categorized into 2 groups to derive a dietary diversity status for pregnant women. The dietary diversity score of five or more was considered adequate (coded as 1), and the score below five was inadequate (coded as 0) [21, 23].

### 3.3. Ethnicity

A caste/ethnicity classification used by the Health Management Information System (HMIS) of the Ministry of Health and Population, Nepal, was adapted for this study. This system uses six caste groups: Dalits, Janajati, Muslim, Madhesi, Brahmin/Chhetri, and others, of which the first four groups were taken as they are considered belonging to the underprivileged groups. Among Dalit and Janajati, only Terai Dalits and Terai Janajati were present during the period of data collection.

### 3.4. Data Management and Analysis

The data were entered into EpiData Entry version 3.1 and then transferred into IBM SPSS version 23 software for analyses. In the first stage, descriptive analyses were performed. Frequency tables with percentage were generated for categorical variables, while mean and standard deviation (SD) were calculated for continuous variables. In the next stage, bivariate and multivariate analyses were performed to determine the factors associated with anemia. Variables that were significant at 15% significance level in bivariate analyses using Pearson's chi-square test were entered into a multiple logistic regression model [24]. Before performing the regression analysis, a test of multicollinearity was done. One of the variables “gravida” showed multicollinearity (variance inflation factor (VIF) >5) and was excluded from the analysis. Model fit was measured with the Hosmer and Lemeshow goodness-of-fit test; the model was found to be a good fit with *p* > 0.05. Odds ratios (OR) were presented with their corresponding 95% confidence intervals (CIs), and a probability value (*p* value) of less than 0.05 was considered statistically significant.

### 3.5. Ethical Considerations

The study protocol was approved by the Institutional Review Committee of the Institute of Medicine, Tribhuvan University (Reference no. 46(6–11E)2/074/075). The study was fully abided by the ethical guidelines of Nepal Health Research Council. Approval to conduct this study was also obtained from Janakpur Provincial Hospital (previously known as Janakpur Zonal Hospital). All participants were above the age of 18. A written informed consent (in Nepali language) was obtained from the participants before the interview and blood sample collection. In case of illiterate participants, the consent form was read out and thumb impressions were obtained in the presence of a literate witness. The participation in the study was voluntary, and no incentives were provided. Also, the participants did not have to pay any charges for laboratory tests. Furthermore, the study participants had the right to withdraw from the study at any time. Personal identifier was omitted in the questionnaire to maintain anonymity and to ensure confidentiality of information.

## 4. Results

### 4.1. Characteristics of the Study Population

The age of the women ranged from 18 to 37 years. The mean age of the respondents was 22.6 years (SD = 3.9 years). Majority of the women were in the age group 20–24 years (45.6%), were Madhesi (65.2%), and followed Hinduism (90.2%). Only one-fourth of pregnant women (27.2%) had completed their secondary level education. Greater majority of the pregnant women lived in joint and extended families (81.5%) and were homemaker (89.9%). At the time of the interview, 41.5 and 58.5 percent were in their second and third trimesters of pregnancy, respectively. More than half (53.7%) of the pregnant women were multigravida. Among the multigravida women, more than one-fourth (26.6%) had a birth interval of more than two years. About one in ten (9.4%) pregnant women had a history of miscarriage/abortion (Table 1).

### 4.2. Preventive Health Practices

In our study, only about a fourth of pregnant women (28.8%) had their four ANC visits completed. More than four in five women had consumed iron-folic acid (88.1%) and deworming medicine (81.8%). All pregnant women (100%) used mosquito net while sleeping (Table 2).

### 4.3. Dietary Practices

The starchy staple foods were consumed by all pregnant women (100%). A greater majority of the women consumed pulses (97.6%), dairy products (90.9%), and other vegetables (84.0%). About half of the respondents consumed other fruits (52.6%) while less than half consumed dark green leafy vegetables (44.3%), meat, poultry, and fish (30.3%), and eggs (15.7%). One-fifth (21.9%) consumed other vitamin A-rich fruits and vegetables while only one-tenth (11.5%) consumed nuts and seeds. More than two-thirds of the respondents (69.3%) had adequate dietary diversity as they consumed at least five of the ten food groups. More than 1 in 10 women (15.7%) avoided certain food groups in pregnancy for cultural reasons (Table 3).

### 4.4. Prevalence of Anemia

The overall prevalence of anemia (hemoglobin <11.0 g/dl) was 66.9% (95% CI, 60.3%–71.2%). In terms of severity, mild anemia was 64.8% (95% CI, 59.0%–70.3%), moderate anemia was 1.7% (95% CI, 0.57%–4.02%), and one woman was severely anemic (Table 4).

### 4.5. Factors Associated with Anemia

The anemia status of pregnant women was compared with sociodemographic characteristics, preventive health practices, and dietary diversity status. In the bivariate analyses, a statistically significant association was found with ethnicity (*p* < 0.01), religion (*p*=0.008), education (*p* < 0.001), place of residence (*p*=0.019), gravida (*p*=0.006), frequency of antenatal visits (*p* < 0.001), consumption of iron-folic acid (*p*=0.032) and deworming medicines (*p*=0.001), and dietary diversity status (*p* < 0.001). However, the association was not statistically significant for age, occupation, family type, pregnancy trimester, birth interval, history of miscarriage/abortion, and practice of food avoidance (Table 5).

The regression analysis showed that the anemia status in pregnant women was significantly associated with ethnicity, education, history of consumption of deworming medicines, frequency of antenatal visits, and dietary diversity status. The odds of anemia were higher among pregnant women of underprivileged ethnic group and those with lesser education. The underprivileged women such as Terai Dalit, Janajati, and Muslims were 2 times more likely to be anemic (AOR, 2.34; 95% CI, 1.06–5.17) compared to Madhesis. Similarly, those with education below secondary level were about 3 times more likely to be anemic (AOR, 2.87; 95% CI, 1.52–5.45) than those having at least secondary level education. It was also observed that the pregnant women who did not complete their four antenatal visits were two times more likely to be anemic (AOR, 2.28; 95% CI, 1.23–4.22) than their counterparts who had completed all four visits. Pregnant women who had not consumed deworming medicines were three times more likely to be anemic (AOR, 3.03; 95% CI, 1.20–7.65) that those who had consumed the tablets. Furthermore, women who did not have adequate dietary diversity were four times more likely to be anemic (AOR, 4.25; 95% CI, 1.81–9.98) compared to women having adequate diversity in their diet (Table 6).

## 5. Discussion

In the present study, two in three pregnant women (66.9%) were anemic, signifying a severe public health problem [25]. This figure is more than two times higher than the prevalence reported among pregnant women from midwestern Nepal (28.3%) [26]. The anemia prevalence in our study is also higher than both the national (41%) and provincial estimates (58%) for the women of reproductive age [6]. The discrepancies in the prevalence might be due to hospital-based study setting, inclusion of only the women from underprivileged ethnic groups, different study periods, and regional variations in the socioeconomic status and dietary practices. Being a referral level hospital, it is also possible that some of the pregnant women attending ANC at the study hospital were referred from peripheral health facilities after being suspected for pregnancy-related complications including anemia. However, such information was not explored in this study. The anemia prevalence in our study was comparable with the study conducted among pregnant women in a similar setting [27]. The PoSHAN community studies' baseline report had also reported similar findings among the pregnant women in the Terai region [28].

In our study, the factors associated with anemia among underprivileged pregnant women were ethnicity, education, intake of deworming medication, antenatal visits, and dietary diversity status. Based on the available evidence from Nepal, it is clear that pregnant women from underprivileged or disadvantaged ethnic groups are more likely to be anemic compared to the upper caste groups. Our study further revealed that the burden of anemia is unevenly distributed even within the underprivileged ethnic groups. The odds of anemia in pregnancy were two times higher among the most-marginalized groups (Terai Dalit, Terai Janajati, and Muslims) compared to Madhesi women. This finding is quite obvious as Terai Dalits and Muslims occupy the lowest position in the human development and poverty indices [29, 30] and suffer caste-based discriminations much higher than other groups. The underlying structural factors such as poor literacy, caste-based discriminations, lesser autonomy in decision making, and other cultural restrictions might have predisposed these women towards greater anemia risks. Furthermore, both the per capita consumption expenditure and land ownership are reported to be lowest among the Terai Dalit caste groups [31], suggesting their limited capacity to consume adequate and diverse foods. This finding warrants the need for special targeting to women from the most disadvantaged ethnic groups with nutritional programs and interventions in order to “leave no one behind” as pledged in the 2030 agenda [14].

Our study found significantly higher odds of anemia among pregnant women with lower education levels. Women who had education below the secondary level were about three times more likely to be anemic than others. This is consistent with the findings of similar studies where anemia was inversely associated with maternal education [32–35]. This is perhaps due to the benefits associated with education. For example, higher education can contribute to higher productivity and earnings which in turn might have positive influences on women's dietary practices. Studies have also documented the positive impact of educational attainment on the quality of women's diet [36]. Women's education might also be associated with women's autonomy and empowerment. Autonomous women are likely to obtain more information and make better decisions regarding their nutrition, improve healthcare seeking, and influence intrahousehold food distribution [37, 38]. Benefits of completing secondary education on the nutritional status of women must therefore be recognized.

Low dietary diversity significantly predicted anemia in our study population. Pregnant women who did not have adequate diversity in their diet were four times more likely to be anemic compared to women whose dietary diversity was adequate. A number of previous studies from Nepal and other LMICs have also confirmed the association of low dietary diversity with anemia [39–42]. The role of dietary diversity in ensuring the adequate hematological status of pregnant women and thus its contribution in reducing the likelihood of anemia has been well documented [43]. Nonetheless, the anemia prevalence in our study was higher although more than two-thirds of pregnant women were found to have an adequate dietary diversity. This might be because although the pregnant women reported consuming items from diverse food groups, they might have been consumed in low frequency and small portion sizes [28, 44–46]. Women often suffer the most from inequitable intrahousehold food distribution and are often the last in the household serving order [44–46]. Furthermore, one study conducted among Tharu and Musahar populations (indigenous groups of Terai) revealed that the diet in Terai is dominated by rice (cereals) with only tiny portions of side dishes such as lentils and vegetables which are insufficient to meet the recommended dietary allowance for micronutrients [47]. Although green vegetables and other diverse foods are readily grown and available in the Terai region, people do not regard them as food items that should be consumed in large amounts [47]. This highlights the need for strong targeted behavior change communication (BCC) intervention that promotes not only dietary diversity but also the nutritional value of locally available foods and need for optimal intake of diets from each group (dietary adequacy). Additionally, the possibility that lower dietary diversity in our study population might have been influenced by household food insecurity cannot be undermined. Results of national survey data found significantly higher odds of experiencing household food insecurity by Dalit women [48]. The interventions to reduce micronutrient deficiency in the region should therefore aim to address dietary diversity, dietary adequacy, and food security targeting the ethnically disadvantaged populations.

Evidence shows that low coverage of deworming medications during pregnancy results in increased parasitic infections [49, 50] and is associated with higher rates of anemia in pregnant women. Not having a deworming medication was one of the associated factors among our study population. Respondents who had not taken deworming medicines in their current pregnancy had three times higher odds of anemia than their counterparts. This is comparable with the evidence from previous studies [41, 51, 52]. Further in our study, fewer ANC visits were associated with increased chances of anemia among the study population. This can be explained by the fact that late and infrequent ANC visits might deny women or delay the provision of iron-folic acid supplementation, deworming medication, and/or malaria prophylaxis whereas women who seek ANC frequently are more likely to benefit from counseling and advices concerning nutrition, preventive health behaviors, and healthful dietary practices. Our finding is consistent with previous studies which have reported an association between infrequent ANC visits and anemia [32, 41, 53]. Promoting the coverage and frequency of antenatal visits is therefore considered vital to reducing anemia in pregnant women.

There are some notable limitations which must be taken into account while interpreting the results of the study. First, the cross-sectional nature of our study makes it difficult to establish the temporal relationships. Second, the assessment of dietary diversity status was based on 24-hour dietary recall, which may not always represent the actual intake. Moreover, the possibility of recall bias also cannot be ruled out completely. Third, our prevalence estimate was based on participants enrolled in the hospital-based setting, which may differ from that of community-based studies. Lastly, it is important to recognize that underprivileged ethnic groups from hill/mountain such as hill Dalit and hill Janajati could not be represented in this study as they were not present at ANC during the data collection period.

## 6. Conclusions

The prevalence of anemia is a severe public health problem among pregnant women of underprivileged ethnic groups in Province 2. Being Dalit, Janajati, and Muslim, having lower education, less frequent antenatal visits, not receiving deworming medication, and having inadequate dietary diversity are found to be the significant factors. The present study highlights the dire need of improving the frequency of antenatal visits and coverage of deworming program in ethnic populations. Furthermore, promoting a dietary diversity at the household level would help lower the prevalence of anemia. The study findings also imply that the nutrition interventions to control anemia must target and reach pregnant women from the most-marginalized ethnic groups and those with lower education.

## Figures and Tables

**Table 1:** Sociodemographic characteristics of the study population (*n* = 287).

Variables	Number	Percent
*Age group* (*years*)^a^		
≤19	64	22.3
20–24	131	45.6
25–29	66	23.0
≥30	26	9.1

*Ethnicity*		
Madhesi	187	65.2
Terai Dalit	55	19.2
Muslim	29	10.1
Terai Janajati	16	5.5

*Religion*		
Hindu	259	90.2
Muslim	28	9.8

*Educational status of the respondent*		
Below secondary level	209	72.8
Secondary level and above	78	27.2

*Family type*		
Joint/extended	234	81.5
Nuclear	53	18.5

*Occupation*		
Homemaker	258	89.9
Employed (job, business, and labor)	29	10.1

*Place of residence*		
Urban	214	74.6
Rural	73	25.4

*Pregnancy trimester at interview*		
Second	119	41.5
Third	168	58.5

*Gravida*		
Primigravida	133	46.3
Multigravida	154	53.7

*Birth interval* (*n* *=* 154)		
≤2 years	113	73.4
>2 years	41	26.6

*History of miscarriage/abortion*		
No	260	90.6
Yes	27	9.4

^a^Mean ± SD = 22.6 ± 3.9.

**Table 2:** Preventive health practices (*n* = 287).

Variables	Number	Percent
*Number of antenatal visits*		
<4 times	206	71.8
≥4 times	81	28.2

*Consumed iron-folic acid*		
Yes	252	87.8
No	35	12.2

*Consumed deworming medicine*		
Yes	234	81.5
No	53	18.5

*Used mosquito net while sleeping*		
Yes	287	100

**Table 3:** Dietary practices of the pregnant women (*n* = 287).

Variables	Number	Percent^a^
*Food groups*		
Starchy staples (grains, white roots, and tubers)	287	100.0
Pulses (beans, peas, and lentils)	280	97.6
Nuts and seeds	33	11.5
Dairy	261	90.9
Meat, poultry, and fish	87	30.3
Eggs	45	15.7
Dark green leafy vegetables	127	44.3
Other vitamin A-rich fruits and vegetables	63	21.9
Other vegetables	241	84.0
Other fruits	151	52.6

*Dietary diversity*		
Adequate	199	69.3
Inadequate	88	30.7

*Food avoidance during pregnancy*		
Yes	45	15.7
No	242	84.3

^a^The total adds of percentage are more than 100% as the pregnant women consumed food items from multiple food groups.

**Table 4:** Prevalence of anemia among pregnant women (*n* = 287).

Variables	Number	Percentage	95% CI
Anemia	192	66.9	61.1–72.3

*Severity of anemia*			
Mild anemia	186	64.8	59.0–70.3
Moderate anemia	5	1.7	0.57–4.02
Severe anemia	1	0.3	—

**Table 5:** Association of anemia status with various characteristics.

Sociodemographic characteristics, preventive health practices, and dietary diversity status	Anemic no. (%) (*n* = 192)	Nonanemic no. (%) (*n* = 95)	*p* value
*Age group*			
≤19	45 (23.4)	19 (20.0)	0.297
20–24	82 (42.7)	49 (51.6)	
25–29	44 (22.9)	22 (23.2)	
≥30	21 (10.9)	5 (5.3)	

*Ethnicity*			
Terai Dalit/Janajati/Muslim	86 (44.8)	15 (14.7)	<0.001^a^
Madhesi	106 (55.2)	81 (85.3)	

*Religion*			
Hindu	167 (87.0)	92 (96.8)	0.008
Muslim	25 (13.0)	3 (3.2)	

*Educational status*			
Below secondary level	160 (83.3)	49 (51.6)	<0.001^a^
Secondary level and above	32 (16.7)	46 (48.4)	

*Family type*			
Nuclear	34 (17.7)	19 (20.0)	0.638
Joint/extended	158 (82.3)	76 (80.0)	

*Occupational status*			
Homemaker	177 (92.2)	81 (85.3)	0.067
Employed (job, business, and labor)	15 (7.8)	14 (14.7)	

*Place of residence*			
Urban	135 (70.3)	16 (16.8)	0.019^a^
Rural	57 (29.7)	79 (83.2)	

*Pregnancy trimester at interview*			
Second	85 (44.3)	34 (35.8)	0.127
Third	107 (55.7)	61 (64.2)	

*Gravida*			
Multigravida	114 (59.4)	40 (45.0)	0.006^a^
Primigravida	78 (40.6)	55 (57.9)	

*Birth interval* (*n* = 154)			
≤2 years	88 (77.2)	25 (62.5)	0.70
>2 years	26 (22.8)	15 (37.5)	

*History of miscarriage/abortion*			
No	175 (91.1)	89 (89.5)	0.648
Yes	17 (8.9)	11 (10.5)	

*Number of antenatal visits*			
<4 times	153 (79.7)	53 (55.8)	<0.001^a^
≥4 times	39 (20.3)	42 (44.2)	

*Consumption of iron-folic acid*			
Yes	163 (84.9)	89 (93.7)	0.032^a^
No	29 (15.1)	6 (6.3)	

*Consumption of deworming medicine*			
Yes	146 (76.0)	88 (92.6)	0.001^a^
No	46 (24.0)	7 (7.4)	

*Food avoidance during pregnancy*			
Yes	31 (16.1)	14 (14.7)	0.757
No	161 (83.9)	81 (85.3)	

*Dietary diversity*			
Inadequate	80 (41.7)	8 (8.4)	<0.001^a^
Adequate	112 (58.3)	87 (91.6)	

^a^Statistically significant at *p* < 0.05.

**Table 6:** Multivariate analysis of factors associated with anemia in pregnancy.

Variable	Adjusted OR	95% CI
*Ethnicity*		
Terai Dalit/Janajati/Muslim	2.34	1.06–5.17^a^
Madhesi	1	1

*Religion*		
Muslim	1.59	0.38–6.68
Hindu	1	1

*Educational status*		
Below secondary level	2.87	1.52–5.45^a^
Secondary level and higher	1	1

*Occupational status*		
Homemaker	1.75	0.65–4.73
Employed (job, business, and labor)	1	1

*Place of residence*		
Rural	1.12	0.54–2.32
Urban	1	1

*Frequency of antenatal visits*		
<4 times	2.28	1.23–4.22^a^
≥4 times	1	1

*Consumption of iron-folic acid tablets*		
No	1.71	0.62–4.73
Yes	1	1

*Consumption of deworming medicines*		
No	3.03	1.20–7.65^a^
Yes	1	1

*Dietary diversity*		
Inadequate	4.25	1.81–9.98^a^
Adequate	1	1

^a^Statistically significant at *p* < 0.05.

## Data Availability

The datasets generated during the current study are available from the corresponding author on reasonable request.

## Abbreviations

ANC: Antenatal care
OR: Odds ratio
SD: Standard deviation
SPSS: Statistical Package for Social Sciences
WHO: World Health Organization.
